# Editorial: Medicinal plants as a source of novel autoimmune-modulating and anti-inflammatory drug products

**DOI:** 10.3389/fphar.2022.978581

**Published:** 2022-09-06

**Authors:** Yang Wang, Ji Chen, Jun Tian, Yihai Wang, Zhengang Zha, Xiaobin Zeng

**Affiliations:** ^1^ Central Laboratory of Longhua Branch and Department of Infectious Disease, Shenzhen People’s Hospital (The Second Clinical Medical College, Jinan University, The First Affiliated Hospital, Southern University of Science and Technology), Shenzhen, China; ^2^ School of Pharmaceutical Sciences (Shenzhen), Shenzhen Campus of Sun Yat-sen University, Shenzhen, China; ^3^ Department of Orthopaedics, Shenzhen People’s Hospital (The Second Clinical Medical College, Jinan University, The First Affiliated Hospital, Southern University of Science and Technology), Shenzhen, China; ^4^ College of Life Science, Jiangsu Normal University, Xuzhou, China; ^5^ School of Pharmacy, Guangdong Pharmaceutical University, Guangzhou, China; ^6^ Department of Orthopaedics, The First Clinical Medical College, Jinan University, Guangzhou, China; ^7^ Guangdong Provincial Key Laboratory of Regional Immunity and Diseases, Medicine School of Shenzhen University, Shenzhen, China

**Keywords:** functional foods, autoimmune diseases, inflammatory diseases, pathogenesis, drug development, medicinal plants

## Medicinal plants as a source of novel autoimmune modulating and anti-inflammatory drug products

Inflammation response is activated as the first line of defense against the invasion of foreign pathogens and harmful stimuli, which in turn helps the body repair damaged tissue and remove irritants and damaged cells. Basically, Cytokine mediate the occurrence of an effective immune response, although cytokines are well initiated and coordinated in immune response, but unregulated cytokine signaling inadvertently constitutes the major determinant of immune pathology, leading to autoimmune diseases or chronic inflammation that affects older people around the world (Bello et al., 2018; Galindo et al., 2018). Such as arthritis (osteoarthritis, rheumatoid arthritis), allergies, atopic dermatitis, psoriasis, asthma, chronic obstructive pulmonary disease, inflammatory bowel diseases, steatohepatitis (Placha and Jampilek, 2021). The classic anti-inflammatory drugs are glucocorticoids and cyclooxygenase (COX-1 and 2) inhibitors. However, withdrawn COX-2 inhibitors, such as valdecoxib and rofecoxib, were associated with a higher risk of stroke, while etoricoxib raised blood pressure (Davies, 1995). Historically, medicinal plants and natural products have been used in anti-inflammatory treatment, and many new medicines have been discovered from herbal sources, most of which are plant-derived secondary metabolites (Akhtar, 2022). Such as quercetin and kaempferol (Hämäläinen et al., 2007) and resveratrol (Kundu et al., 2006). Therefore, it is a very important strategic approach to seek effective anti-inflammatory inhibitors from medicinal plants.

Macrophages play a key role in “inflammatory pathways” that lead to a range of diseases and disorders (Chen et al., 2018). The classical activation of macrophages occurs after injury or infection of microbial products or pro-inflammatory cytokines, including bacterial lipopolysaccharide (LPS), interferon-γ (IFN-γ), or tumor necrosis factor-α (TNF-α), and trigger inflammatory signaling pathways, most commonly, NF-Kb (nuclear factor kappa-light-chain-enhancer of activated B cells), MAPK (mitogen-activated protein kinase) and JAK-STAT (Janus kinases (JAKS)—signal transducer and activator of transcription proteins) (Besednova et al., 2022). Recently, the non-classical Hippo pathway and NLRP3 inflammasome have been highlighted in inflammatory responses of macrophage (Jo et al., 2016; Tsai et al., 2021; Xie et al., 2021). However, the molecular mechanisms underlying autoimmunity regulation or anti-inflammatory potential remain unclear. Therefore, it has become a research hotspot to explore the molecular mechanism of autoimmunity regulation or anti-c and discover new therapeutic schemes.

The 13 articles collected in this Research Topic focus on the most recent advances on medicinal plants as a source of novel autoimmune modulating and anti-inflammatory, mainly on novel molecular targets and on innovative pharmacological approaches. Kangfuxiaoyanshuan (KFXYS) is a traditional Chinese herbal compound that composed of eight classical medicinal herbs. The various chemical components of this Chinese herbal compound, including alkaloids, flavonoids, diterpenoid lactones, bile acids, anthraquinones, naphthoquinones, and organic acids (Xie et al., 2019). Here, Liu et al. demonstrate KFXYS remarkablely reduced the levels of proinflammatory factors (IFN-31γ, IL-1β, IL-4) and adhesion related factors (TNF-α) in the acute stage of pelvic inflammatory disease (PID), and had protective effect of ultrastructure of endometrial epithelial cells. In addition, KFXYS had been exerted significant anti-inflammatory effect though inhibiting the activation of the NF-κB pathway, and decreasing the TGF-β/MMP-2 associated tissue adhesion. Gao et al. reported that salvianolate (total polyphenols), a traditional Chinese medicine extracted from Salvia miltiorrhiza, ameliorated osteopenia and bone quality in glucocorticoid treated RA rats. Combined treatment with prednisone and salvianolate for ameliorating joint damage and improved bone quality in collagen-induced arthritis rats via activation of the RANKL/RANK/OPG signaling pathway and TRAIL-TRAF6-NFκB signal axis, thus decreasing the risk of fracture. The study by Tu et al. showed that the alleviating inflammatory responses and cartilage degradation of Rhoifolin (a flavanone extracted from Rhus succedanea) in rat chondrocytes is mediated by P38/JNK and PI3K/AKT/mTOR signal pathways.

NLRP3, a sensor of innate immunity, mediates the activation of caspase-1 and secretion of pro-inflammatory cytokine IL-1β/IL-18 through the formation of a complex called inflammasome. Abnormal activation of the NLRP3 inflammasome has been associated with a variety of inflammatory disorders, including metabolic disorders, gout, neurodegenerative diseases and autoimmune diseases. Therefore, suppressing the activity of NLRP3 inflammasome becomes a novel signaling pathway on preventing and treating inflammatory diseases. *Artemisia argyi H. Lév. and Vaniot* is a traditional herb medicine that has been widely applied for treating inflammation-related diseases. Chen et al. demonstrated that essential oil of *Artemisia argyi H. Lév. and Vaniot* (EOAA) inhibited the activation of NLRP3 inflammasome in THP-1 cells induced by monosodium urate and nigericin. Feng et al. confirmed that ethyl 2-succinate-anthraquinone (LHD) has better anti-inflammatory and antibacterial activity than aloe emodin, a novel compound has been chemically modified. It has been an effect on anti-inflammatoion effects through reducing the expression of NLRP3, IL-1β, and caspase-1 protein thus mediating the NLRP3 inflammasome signaling pathway.

Many plants with autoimmune modulatory or anti-inflammatory potential remain unexplored. It is high time that these medicinal plants and natural products were systematically studied using modern tools. For example, the prediction and putative targets of traditional Chinese medicine prescriptions on the mechanism of autoimmune regulation or anti-inflammatory action were further analyzed based on network pharmacology, and further enrichment analysed by KEGG pathways and GO terms related with biological processes, molecular functions, and cellular components. New autoimmune modulatory or anti-inflammatory plant products can also be discovered through high-throughput and high-content screening. In the meanwhile, to develop an immunocompetent 3D *in vitro* model allows screening of potential plant derived therapeutic agents against inflammation.

Rheumatoid factor (RF) or anti-cyclic citrullinated peptide antibodies (anti-CCP) are serological indicators for the diagnosis of RA. Through meta-analysis and systematic review, Tang et al. found that the treatment of RA by Chinese herbal compound could effectively reduce the level of RF or anti-CCP, and the clinical efficacy might be superior to western single drug therapy. They summarized the active components and pharmacological effects of five high-frequency representative Chinese herbal medicines, including *Angelica sinensis* (Oliv.) Diels, *Glycyrrhiza glabra* L., *Neolitsea cassia* (L.) Kosterm., *Paeonia lactiflora* Pall., and *Angelica dahurica* (Hoffm.) Benth. and Hook. f. Ex Franch. and Sav., and found that Chinese herbal compound may reduce RF and anti-CCP levels by regulating immune response and inhibiting B lymphocyte proliferation. Through network pharmacology, Huang et al. revealed that *Cornus officinalis* Sieb. And *Paeonia lactiflora* Pall. Have been identified as therapeutic candidates to treat RA, as well as their main active compounds ursolic acid and paeoniflorin could reduce synovial hyperplasia and inflammatory infiltration of joint tissues while promoting synovial apoptosis in the rat model of collagen-induced arthritis (CIA).

The “multi-target” feature of traditional Chinese medicine has a paticular advantage in the treatment of inflammatory diseases. The use of specific Chinese herbal medicine or plant compounds can reduce symptoms and pathological damage and play a synergistic healing effects when combined with western medicine. Wang et al. selected five key targets, namely JAK3, RORC, PRKCQ, ZAP70, and IL6, and their corresponding active components and positive drugs as ligands through the network pharmacology method. The interaction between the main active components and potential targets of volatile oil from *Matricaria recutita* L. was verified by molecular docking. *Prunella vulgaris* L. is a traditional Chinese medicine, which has been used to thyroid diseases in China. Xu et al. investigate the bio-active ingredients and mechanisms against hashimoto’s thyroiditis via network pharmacology and molecular docking technology. The key targets of *Prunella vulgaris* L. were analyzed, and the main targets were JUN, AKI, MAPK1, TP53. Molecular docking results suggest that luteolin and kaolin may play an important role in the treatment of hashimoto’s thyroiditis by regulating multiple signaling pathways. Marescotti et al. focuse on the development of an immunocompetent 3D *in vitro* triculture intestinal model contain with a differentiated intestinal epithelial layer and immune-competent macrophages. The model imitated a healthy intestine with stable barrier integrity. They evaluated the model with known anti-inflammatory compounds and natural alkaloids of plant origin. By catching the critical specialty of the intestinal mucosa, the immunocompetent 3D *in vitro* model could select potential plant derived of compounds or extract agents against intestinal inflammation.

The clinical application of Traditional Chinese Medicine (TCM) is one of the most effective complementary drugs for the treatment of autoimmune diseases or inflammatory diseases. However, the molecular therapeutic mechanism of TCM is still unclear, which limits its application worldwide. Xiao-Yin-Fang (XYF) has been used by Shanghai Changhai Hospital to treat patients with psoriasis for more than 30 years, and its efficacy has been proved by clinical studies. Zhang et al. demonstrated that XYF relieved psoriasis-like skin inflammation chiefly by inhibiting polarization of γδT17 cells in dermis and draining lymph nodes. In addition, XYF can improve the recurrence of psoriatic dermatitis and inhibit the activation of γδT cells. Transcriptional analysis showed that XYF may regulate a variety of inflammatory signal transduction and metabolic processes. Here, Jantrapirom et al. reviewed phytochemicals constituents of Triphala, a potential herbal candidate for the treatment of allergic rhinitis. Though inhibiting the phosphorylation of p38, c-Jun N-terminal kinase (JNK), and ERK and activating the Akt/AMP-activated protein kinase/nuclear factor erythoid 2-related factor (Akt/AMPK/Nrf) signaling pathway to reduce NF-κB activation and nuclear translocation. Traditional Chinese Medicine Xuanfei Baidu Decoction (XFBD) has been successfully used in the treatment of COVID-19 in China. Yang et al. evidenced that XFBD has multiple positive effects on enhancing cellular immunity via regulating the immune systems in cyclophosphamide treated mice. Previously, network strategies were used to predict the potential signaling pathways that XFBD may participate in the anti-COVID-19 effect (Wang et al., 2020). XFBD significantly inhibited the expression of IL-6, IP-10, and TNF-α in LPS-induced THP-1 cells, and inhibited the secretion of TNF-α, IL-6, and IL-1β in RAW 264.7 cells, suggesting that XFBD had bidirectional immune modulatory effects.

In summary, this Research Topic provided a platform for medicinal plants researchers to present novel findings on therapeutic targets for inflammation disease and to learn new insights into modern tools between inflammation therapy and medicinal plants. New therapeutic strategies are needed to reduce the harmful effects of chronic inflammation disease. Further research is necessary to understand autoimmune diseases, or inflammatory diseases potential of medicinal plants.
